# “On-Water” Catalyst-Free Ecofriendly Synthesis of the Hantzsch Dihydropyridines

**DOI:** 10.5402/2012/342738

**Published:** 2012-08-12

**Authors:** Amit Pramanik, Manabendra Saha, Sanjay Bhar

**Affiliations:** ^1^Department of Chemistry, Taki Government College, North 24 Pgs 743 429, India; ^2^Department of Chemistry, Surendranath Evening College, Kolkata 700 009, India; ^3^Department of Chemistry, Jadavpur University, Kolkata 700 032, India

## Abstract

An eco-friendly “on-water” protocol for efficient catalyst-free synthesis of the Hantzsch dihydropyridines from aryl, heteroaryl, alkyl, and vinylogous aldehydes has been developed with minimum auxiliary substances, toxic reagents, organic solvents, and disposal problems.

## 1. Introduction

4-Substituted 1,4-dihydropyridine framework has emerged as one of the most prolific chemotypes in the recent computational analysis of medicinal chemistry database due to its multifarious pharmaceutical applications. They serve as important analogues of NADH coenzymes [1] exhibiting neuroprotectant [2] and platelet anticoagulatory activity [3]. These compounds often act as cerebral anti-ischemic [3] agents in the treatments of Alzheimer's disease and as chemosensitizers [4] in tumour therapy. Due to their high efficiency as Ca^2+^ channel blockers, the Hantzsch dihydropyridines also find immense applications in the treatment of cardiovascular disorders and hypertension [5]. 1,4-Dihydropyridine skeleton is also present in many vasodilator, bronchodilator, antiatherosclerotic, antitumor, antidiabetic, geroprotective, and hepatoprotective agents. Moreover, these compounds serve as important synthetic intermediates [6, 7] for the preparation of various pyridine derivatives through oxidative aromatization sequences. A number of synthetic protocols for the construction of the dihydropyridine skeleton are available in the literature using ammonia [8], refluxing ammonium hydroxide in a closed vessel microwave synthesizer [9], urea-silica gel [10], ammonium acetate in ethanol under microwave irradiation [11], ammonium hydroxide in ethanol [12], 2,4,6-trichloro-1,3,5-triazine [13], magnesium nitride [14] in water at an elevated temperature in a sealed vessel using stoichiometric excess of organic reactants, and many others. Many of the aforesaid protocols use expensive and toxic reagents (often in excess amounts than required for reaction stoichiometry), have complicated reaction setup, require long reaction times, and form byproducts due to various side reactions. Often these reactions are performed in various organic solvents posing a serious threat of fire hazard, especially when they are carried out under microwave irradiation. Several solvent-free protocols [10] have been developed using supported reagents, but still they require toxic organic solvents during product isolation. Also the disposal of the left-over inorganic supports remains problematic [10] which causes perturbation in the environment. In recent times, ammonium acetate has been judiciously utilized [15, 16] as a convenient source of ammonia during the construction of various important heterocyclic skeletons. Its application for the synthesis of 1,4-dihydropyridines in combination with various reagents like trimethylsilyl iodide [17], tetrabutylammonium hydrogen sulfate in diethylene glycol [18], baker's yeast [19], *p*-toluenesulfonic acid-sodium dodecyl sulphate [20], phenylboronic acid [21], triphenylphosphine [22], and many others has been reported. Many of the aforesaid protocols have limited applicability to a few specific simple aliphatic aldehydes [19] and mostly aryl aldehydes [17, 18, 21, 22] exclusively. Moreover, the aforesaid protocols have some drawbacks in terms of lack of reproducibility, formation of considerable amount of byproducts, use of auxiliary reagents (as promoters) involving separation procedures [17, 18, 21, 22], utilization of inorganic support [18] causing disposal issues, and substantial involvement of organic solvents [17–22] for the isolation and purification of the products through their separation from the auxiliary reagents. Therefore, a better alternative for the synthesis of 1,4-dihydropyridine skeleton in an ecologically benign medium preventing waste [23] and avoiding auxiliary substances [23] is always in great demand in order to improve environmental performance.

## 2. Results and Discussion

**Scheme 1:**
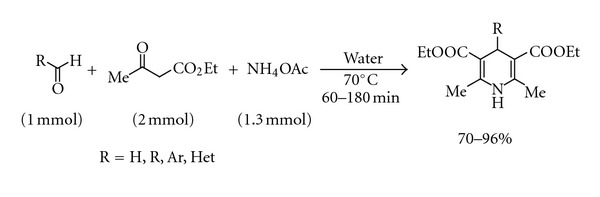


Water is the most abundant environmentally friendly solvent in nature and works as a unique solvent for biochemical processes. Water is also a desirable solvent for *in vitro* chemical reactions from the standpoint of cost and safety. Study of various organic reactions in aqueous medium has emerged as an important arena of contemporary research [24–30]. After the pioneering paper by Sharpless and co-workers [31], numerous synthetic applications of “on-water” reactions have been reported [31–35]. As a part of our ongoing endeavour to develop cost-effective ecofriendly methodology for the construction of important molecular frameworks, we report herein ammonium acetate-mediated catalyst-free “on-water” protocol (Scheme 1) for the synthesis of Hantzsch dihydropyridines bearing various substituents at the 4-position.

Detailed results are presented in Table 1 (relevant reference(s) of the known products are shown in the parenthesis after the literature melting point of the respective product).

**Scheme 2:**
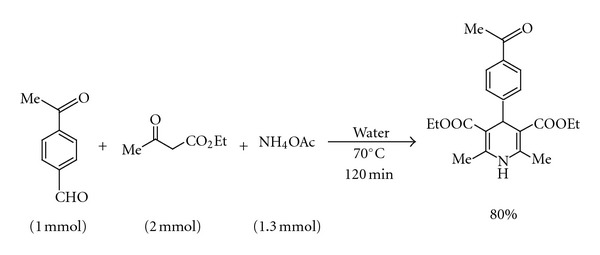


 According to the Table 1, several alkyl, aryl, and heteroaryl aldehydes smoothly underwent rapid “on-water” multicomponent condensation with ethyl acetoacetate and ammonium acetate to accomplish diversely substituted functionally important Hantzsch dihydropyridines in good yield and purity. Aryl aldehydes with electron-donating and electron-withdrawing substituents at various positions furnished the corresponding products without affecting the substituents and the substitution patterns (entries 1–8). Highly vulnerable thermolabile heteroaryl aldehydes also underwent the multicomponent transformation preferentially over thermal polymerization (entries 9 and 10). Interestingly, the *α*, *β*-unsaturated aryl aldehydes underwent clean transformation (entries 11 and 12) without polymerization and other usual side reactions. The analogous *α*, *β*-unsaturated alkyl aldehyde (entry 13) and long-chain saturated alkyl aldehyde (entry 15) reacted better in 1 : 1 ethanol-water and furnished the respective products in moderate yield. Paraformaldehyde, a solid synthetic equivalent of formaldehyde, produced the 4-unsubstituted dihydropyridine (diludine) in good yield (entry 14) under “on-water” condition. This compound has important applications in metal-free transfer hydrogenation reactions [36]. It shows antioxidant activity in *β*-carotene-methyl linoleate, sunflower oil, and emulsion [37] and acts as inhibitor of peroxidation of egg yolk lecithin liposome [38]. So the reported method can be utilized for the rapid and efficient synthesis of the Hantzsch dihydropyridine skeletons with a wide range of structural diversity from appropriate aldehydes. The reactions showed excellent chemoselectivity towards aldehydes. This was evident from the fact that the keto moiety remained totally unaffected in an intramolecular competition experiment (Scheme 2).

It is important to note that the byproducts of this reaction (water and acetic acid) are environment benign compared to those of many alternative procedures. The aforesaid protocol completely eliminates the inorganic support and toxic and flammable organic solvents as reaction medium, uses water as the ecologically most accepted reaction medium, and utilizes ecocompatible organic solvent, namely, ethanol, in small amount during workup in most of the cases. Moreover, unlike the previous procedures [17–22], the present “on-water” protocol does not require any auxiliary reagent as catalyst. Therefore, separation of the product from the auxiliary substance and subsequent purification is totally eliminated. This simplifies the entire procedure making it more cost effective. Also the problem of disposal of the leftover auxiliary is totally eliminated. The pH of the water left after isolation of the product was found to be 5.86. 30 mL of this water, after evaporation, produced 31 mg of residue, mainly consisting of unreacted ammonium acetate. This water was boiled with charcoal, and the pH was found to be 5.90. After evaporation of 40 mL of charcoalized water, 29 mg of residue was left. 

Although 1 : 1 ethanol-water combination gave better results with alkyl (conjugated and saturated) aldehydes (entries 13 and 15 in Table 1), yet the majority of the reactions involving poorly water-miscible aryl and vinylogous aryl aldehydes (entries 1 to 12 in Table 1) smoothly occurred under “on-water” condition. It is important to note that the reactions with aryl aldehydes in the presence of ammonium acetate did not work well in 1 : 1 ethanol-water combination and produced numerous unidentified byproducts with very little formation of the desired dihydropyridines (as evident from the TLC and NMR analyses). Therefore, water comes out as the better alternative to 1 : 1 ethanol-water combination. There are reports [39, 40] for the preparation of 4-unsubstituted dihydropyridine (diludine) in refluxing aqueous ethanol (1 : 1) where liquor ammonia and formalin (aqueous solution of formaldehyde) reacted with ethyl acetoacetate and the product was obtained in modest yield (40–60%). It is important to note that, in the present protocol, the aforesaid compound was prepared with better yield (75%) through the reaction of ammonium acetate and paraformaldehyde (a solid synthetic equivalent of formaldehyde) with ethyl acetoacetate under “on-water” condition at room temperature without any cosolvent like ethanol. Moreover, the reported processes [39, 40] utilize liquor ammonia and formalin as the reagents. From the standpoints of safety, toxicity, and ecocompatibility, ammonium acetate (a noncorrosive stable solid source of ammonia having toxicity parameters IPR-RAT LD50 632 mg kg^−1^; IPR-MUS LD50 736 mg kg^−1^ [41]) is more acceptable than liquor ammonia (corrosive, lachrymatory, destructive to mucous membrane, dangerous to environment, and toxic to aquatic organism having toxicity parameters IHL-HMN TCLO 5000 ppm/5 m; IHL-RAT LC50 1000 ppm/4 h; IHL-MUS LC50 4230 ppm/1 h for ammonia [42] and ORL-RAT LD50 350 mg kg^−1^; ORL-MAN LDLO 43 mg kg^−1^; IHL-HMN LCLO 5000 ppm for ammonium hydroxide [43]). Similarly, paraformaldehyde (a solid equivalent of formaldehyde having toxicity parameters ORL-RAT LD50 800 mg kg^−1^; IHL-RAT LC50 1070 mg/m^3^/4 h [44]) is a less toxic and ecologically more compatible alternative of formalin (37% aq. solution of formaldehyde which is extremely lachrymatory and destructive to mucous membranes, upper respiratory tract, eyes, kidneys, and skin and causes heritable genetic damage having toxicity parameters IHL-TCLO HMN 17 mg/m^3^/30 m; ORL-WMN LDLO 108 mg kg^−1^; IPR-MUS LDLO 16 mg kg^−1^; ORL-RAT LD50 100 mg kg^−1^; SKN-RBT LD50 270 mg kg^−1^ [45]). Therefore, it is obvious that the present protocol provides a better and more ecofriendly method for the preparation of diludine in comparison to the reported ones [39, 40] where ammonia and formalin are used in 1 : 1 ethanol-water. Although the reactions of alkyl (conjugated and saturated) aldehydes (entries 13 and 15 in Table 1) in the present method works better in 1 : 1 ethanol-water, yet they utilize ammonium acetate as the reagent which is less toxic than ammonia. In this context, the present method bodes for a new ecocompatibility in comparison to the aforesaid reports [39, 40] where only a limited number of compounds have been synthesized. So the present ammonium acetate-mediated “on-water” protocol possesses widespread applicability, uses reagents of negligible toxicity, involves water as the most ecocompatible reaction medium, and minimizes the dispersal of undesired chemicals in the environment. From this standpoint, it can be called a green technology.

Various ammonium salts like ammonium formate, ammonium chloride, ammonium sulfate, and ammonium oxalate were totally unsatisfactory because they took much longer time for complete consumption of the substrates, but the reaction mixture contained inseparable mixtures of unidentified byproducts due to various side reactions with very little formation of the desired products. Using urea as an alternative source of ammonia, the desired Hantzsch dihydropyridine was formed, but it was contaminated with substantial amount of unidentifiable byproducts. Therefore, ammonium acetate has come out as the most reliable and convenient source of ammonia in this protocol. 

Several organic solvents in place of water have been attempted as the reaction medium for this catalyst-free ammonium acetate-mediated reaction (Table 2). It is obvious from Table 2 that water is the best medium for this reaction in terms of reaction outcome, selectivity, yield, and purity of the products. Therefore, the present ammonium acetate-mediated catalyst-free “on-water” protocol for the efficient construction of differently substituted Hantzsch dihydropyridines is unique of its kind and warrants the efficacy of “on-water” protocol with a step forward to improve environmental performance which is a formidable task in recent times for the synthetic organic chemists all over the globe.

## 3. Conclusions 

A novel, efficient, economically viable and ecologically compatible synthesis of structurally varied Hantzsch dihydropyridine has been accomplished using easily accessible substrates and reagents. Notable features of the present green methodology are (a) use of water as the most ecocompatible reaction medium, (b) high yield of the product with good purity, (c) complete elimination of the toxic solvents, reagents, and inorganic support, (d) avoiding the use of other auxiliary substances, (e) minimum perturbation in the surroundings in terms of disposal of byproducts and other waste products due to their minimum involvement and formation during the reaction, and (f) general applicability accommodating a variety of substitution patterns.

## 4. Experimental Section 

### 4.1. General

All organic solvents used for the synthesis were purchased from SRL, India, and were distilled before use. Meting points were measured by using capillary tube and were uncorrected. IR spectra were recorded on IR instrument (Perkin Elmer) using KBr discs. ^1^H- and ^13^C-NMR spectra were obtained on a Bruker-300 spectrometer (300 MHz) in CDCl_3_ solutions with TMS as internal reference. Mass spectrums were measured on HRMS (Qtof micro YA263). Thin-layer chromatographic separations were performed on pre coated silica gel plates (E. Merck). All solvents were distilled before use. 

### 4.2. General Procedure

A mixture of an aldehyde (1 mmol), ethyl acetoacetate (2 mmol), and ammonium acetate (1.3 mmol) was vigorously stirred in water (2 mL) at 70°C for the stipulated period of time (Table 1) till the completion of the reaction (monitored by TLC). After completion of reaction, few drops of ethanol were added to the reaction mixture (to facilitate granulation of the products) followed by crushed ice. A solid product was obtained which was filtered, washed with water, and crystallized from aqueous ethanol, if needed. When the products were viscous oils (entries 13 and 15 in Table 1), they were isolated through extraction with an ecocompatible solvent, namely, ethyl acetate. If the amount of water was further decreased, vigorous stirring was interrupted and caused inferior reactions. The combination of neat reactants in the absence of water did not produce any product. The references of the known products have been cited in parenthesis after the literature melting points in the right column of Table 1.

### 4.3. Spectral Data of the Unknown Compounds

#### 4.3.1. Diethyl 4-(4′-methoxycinnamyl)-2,6-dimethyl-1,4-dihydropyridine-3,5-dicarboxylate

(Entry 12 in Table 1): ^1^H-NMR (300 MHz, CDCl_3_): *δ* 1.27 (t, 6H, *J* = 6.6 Hz), 2.31 (s, 6H), 3.78 (s, 3H), 4.12–4.23 (m, 4H), 4.58 (d, 1H, *J* = 6.3 Hz), 5.62 (s, 1H), 5.98 (dd, 1H, *J*_1_ = 15.5 Hz, *J*_2_ = 6 Hz), 6.18 (d, 1H, *J* = 15 Hz), 6.78 (d, 2H, *J* = 8.4 Hz), 7.23 (d, 2H, *J* = 9 Hz); ^13^C-NMR (75 MHz, CDCl_3_): *δ* 14.4, 19.4, 36.4, 55.2, 59.7, 101.7, 113.7, 127.3, 127.4, 129.7, 130.6, 144.6, 158.6, 167.6. HRMS observed for [M + Na]^+^ at 408.1787, calculated for C_22_H_27_NO_5_ [M + Na]^+^ at 408.1790.

#### 4.3.2. Diethyl 4-(1′-propenyl)-2,6-dimethyl-1,4-dihydropyridine-3,5-dicarboxylate

(Entry 13 in Table 1): ^1^H-NMR (300 MHz, CDCl_3_): *δ* 1.27 (6H, t, *J* = 7.2 Hz), 1.60 (d, 1H, *J* = 5.5 Hz), 2.29 (s, 6H), 4.09–4.27 (m, 4H), 4.38 (d, 1H, *J* = 6.5 Hz), 5.32–5.37 (m, 1H); ^13^C-NMR (75 MHz, CDCl_3_): *δ* 14.4, 17.8, 19.4, 36.0, 59.6, 102.4, 123.4, 132.9, 144.2, 167.8. HRMS observed for [M + H]^+^ at 294.1702, calculated for C_16_H_23_NO_4_ [M + H]^+^ at 294.1706.

#### 4.3.3. Diethyl 4-(4′-acetylphenyl)-2,6-dimethyl-1,4-dihydropyridine-3,5-dicarboxylate

(Scheme 2): IR (KBr): 3344, 2981, 1725, 1688, 1609, 1366, 1262, 1042, 863, 668 cm^−1^; ^1^H-NMR (300 MHz, CDCl_3_): *δ* 1.21 (t, 6H *J* = 7.1 Hz), 2.32 (s, 6H), 2.54 (s, 3H), 4.04–4.11 (m, 4H), 5.04 (s, 1H), 5.91 (s, 1H), 7.37 (t, 2H, *J* = 9.1 Hz,), 7.81 (dd, 2H, *J*_1_ = 8.3 Hz, *J*_2_ = 2.1 Hz); ^13^C-NMR (75 MHz, CDCl_3_): *δ* 13.5, 14.2, 19.4, 26.5, 39.9, 59.7, 103.4, 128.2, 135.1, 144.4, 153.3, 167.3, 198.04. HRMS observed for [M + Na]^+^ at 394.1631, calculated for C_21_H_25_NO_5_ [M + Na]^+^ at 394.1633.

## Figures and Tables

**Table 1:** Ammonium acetate-mediated “on-water” synthesis of the Hantzsch 1,4-Dihydropyridines.

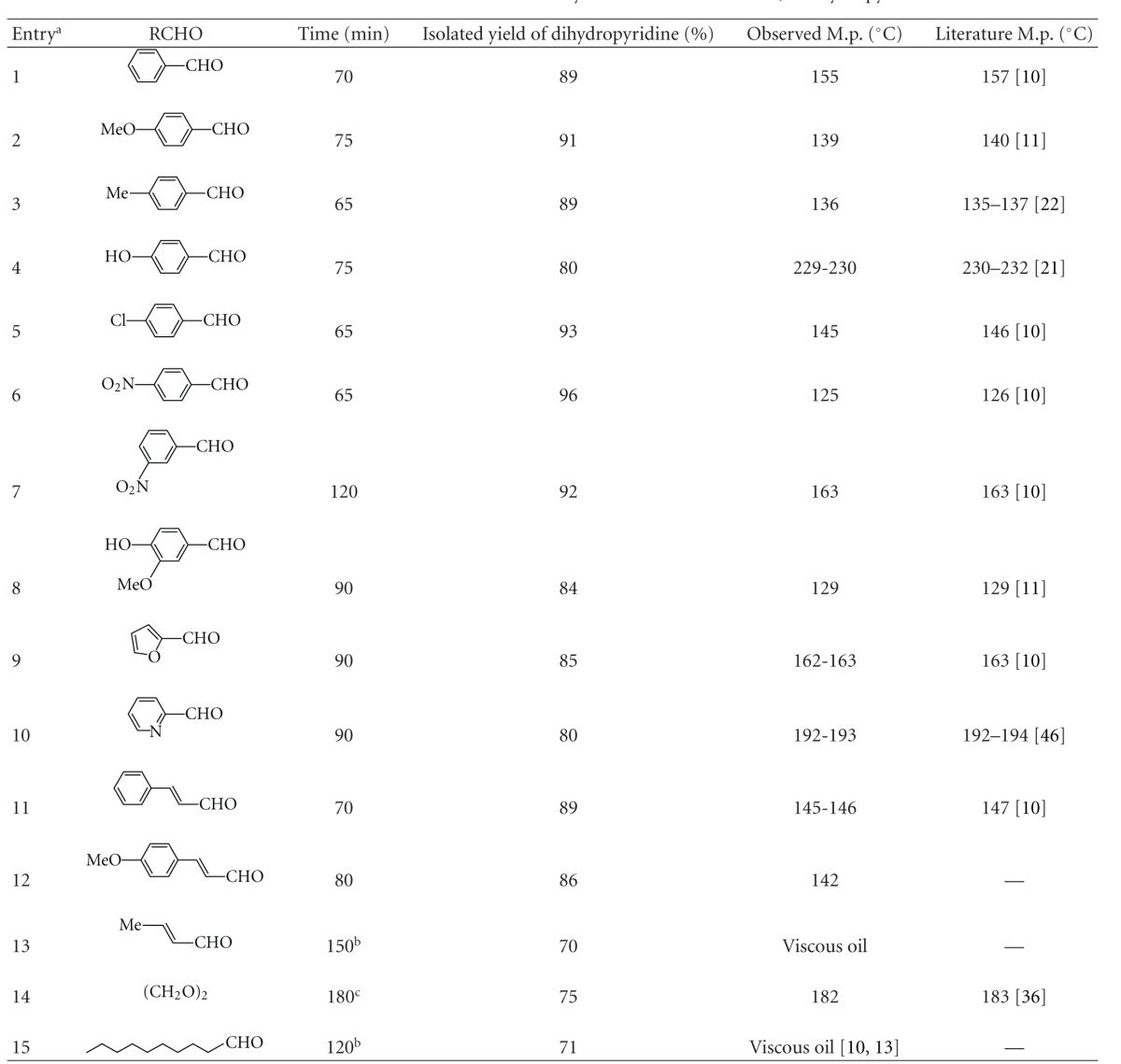

^a^Aldehyde : EAA : ammonium acetate = 1 : 2 : 1.3.

^b^Reactions in water : ethanol = 1 : 1.

^c^Reaction temperature = 30°C.

**Table 2:** Effect of different solvents towards the catalyst-free ammonium acetate-mediated synthesis of the Hantzsch dihydropyridine.

Solvent	Reaction outcome^a^
Hexane	Crude dihydropyridine (58% yield, contaminated with a lot of unidentified byproducts)
Acetone	Mixture of unidentified byproducts without any formation of dihydropyridine
Dichloromethane	Mixture of unidentified byproducts without any formation of dihydropyridine
Chloroform	Mixture of unidentified byproducts with trace amount of dihydropyridine
Ethyl acetate	Crude dihydropyridine (54% yield, contaminated with a lot of unidentified byproducts)
DMF	Exclusive formation of dihydropyridine (44% yield, remainder starting material)
DMSO	Exclusive formation of dihydropyridine (38% yield, remainder starting material)
Ethanol	Crude dihydropyridine (60% yield, contaminated with a lot of unidentified byproducts)
1-Propanol	Crude dihydropyridine (39% yield, contaminated with a lot of unidentified byproducts)
2-Propanol	Crude dihydropyridine (36% yield, contaminated with a lot of unidentified byproducts)
Water	Exclusive formation of dihydropyridine (89% yield of crystallized product) without any formation of byproduct

^a^All the reactions were done with 4-methylbenzaldehyde : EAA : ammonium acetate = 1 : 2 : 1.3 at the boiling point of the corresponding solvent or at 70°C (whichever is lower) for 1 hr.
